# T104. ASSESSING THE UTILITY OF COPULA FUNCTIONS FOR RISK PREDICTION OF PSYCHOSIS

**DOI:** 10.1093/schbul/sby016.380

**Published:** 2018-04-01

**Authors:** Landan Zhang, Charlotte Gayer-Anderson, The EUGEI Study, Craig Morgan, Robin Murray, Michael Pitt, Conrad Iyegbe

**Affiliations:** Institute of Psychiatry, Psychology & Neuroscience, King’s College London

## Abstract

**Background:**

Studies which have attempted to assess the predictive potential of socio-environmental risk factors for psychosis have used such a variety of datasets and methodologies. As a result, it is not possible for policy-makers to understand how different models compare, or might inform evidence-based policy-making. Thus, the cumulative predictive potential of non-genetic risk factors for psychosis has not yet been studied systematically. An important question which has not been considered previously is whether correlation structure between multivariate risks can be detrimental to the goal of prediction, particularly across different populations. Model fitting to locally-relevant correlation structures can limit the generalizability of a prediction model. Copulas are mathematical functions which allow the joint risk function of two or more correlated variables to be modelled in spite of this inherent bias. The copula approach is a foundation methodology with applications in the fields of finance, insurance and banking, where it is used for risk-management purposes. This study examines the impact of copulas on the stability of prediction power for psychosis across different populations.

**Methods:**

The data used in this work comes from work package 2 (WP2) (entitled “Functional Enviromics”) of The European Union (EU)-funded European Network Study of Gene-Environment Interactions (EUGEI). The total dataset available consists of 1180 cases of first episode psychosis (ICD10 diagnostic criteria F20-F29 or F30-F33) and 1528 healthy controls recruited by 16 centres across 6 countries (United Kingdom, Holland, Spain, France, Italy, Brazil). We sought to compare the predictive performance of copulas against that of summary risk scores for formulating disease risk for a common set of socio-environmental risk factors. The copula methodology allows joint risks to be modelled as a distribution whilst summary scores convey the number of risk factors encountered by an individual, weighted by literature-derived odds ratios for association. Gaussian copula with non-Gaussian marginal distributions were used to capture the correlation structure of 9 discrete variables in total. These incorporated: Lifetime Cannabis Use, frequency of Cannabis Use, Household discord, severity of psychological abuse, severity of physical abuse, severity of sexual abuse, severity of bullying, number of adverse adult life events and intrusive adult life events. We applied a fully Bayesian approach which uses Markov Chain Monte Carlo to simulate latent variables from multivariate ordered probit model and also estimate the threshold parameters and parameters from copula model. The resulting joint distribution (a copula) mapped the relationship between cumulative exposure to these factors and risk of psychosis.

**Results:**

A proportion of subjects were withheld from the copula, so that the performance of the finished function could be evaluated on unseen data. The performance of the 2 prediction methods was compared within and between recruitment centres and are conveyed in terms of:

• Sensitivity and false positive rates (The area under the Receiver Operating Characteristic curve)

• Percentage of variance explained (Nagelkerke R2)

• Calibration (whether predicted risks were correct)

• Discrimination (whether high risk subjects could be distinguished from low risk ones)

• Reclassification (model behaviour close to specific thresholds)

**Discussion:**

The application of the copula methodology to the multi-centre EUGEI dataset provides us with the opportunity to tackle a major limitation of the summary scoring approach which is the default method for aggregating risks across most areas of health research.

